# Translation, cultural adaptation and validation of simplified Chinese version of the anterior cruciate ligament return to sport after injury (ACL-RSI) scale

**DOI:** 10.1371/journal.pone.0183095

**Published:** 2017-08-17

**Authors:** Tianwu Chen, Peng Zhang, Yunxia Li, Kate Webster, Jian Zhang, Wei Yao, Yue Yin, Chingchong Ai, Shiyi Chen

**Affiliations:** 1 Department of Sports Medicine and Arthroscopy, Huashan Hospital, Shanghai, China; 2 Sports Medicine Center, Fudan University, Shanghai, China; 3 Musculoskeletal Research Centre, La Trobe University, Victoria, Australia; 4 West Anhui University Health Vocational College, Luan, Anhui Province, China; Mayo Clinic Minnesota, UNITED STATES

## Abstract

**Purpose:**

To translate and cross-culturally adapt the anterior cruciate ligament-return to sport after injury (ACL-RSI) into simplified Chinese [ACL-RSI (Cn)].

**Method:**

In this diagnostic study, the translation, cross-culturally adaptation, and validation of the ACL-RSI was performed according to international guidelines. A total of 112 patients with ACL reconstruction participated in this study. All were capable of competitive sports before the injury and completed the Knee Injury and Osteoarthritis Outcome (KOOS), the International Knee Documentation Committee (IKDC), the Tampa Scale of Kinesiophobia (TSK), and the Tegner activity score. Forty-eight patients completed the ACL-RSI (Cn) twice within two weeks. The validity was tested using seven premade hypotheses. Internal consistency, reliability, and measurement error was assessed.

**Result:**

At meanly 15.6 months postoperative, 81 (72.3%) patients returned to sport, with 57 (50.9%) to competitive sport and 24 (21.4%) to recreational sport. Thirty-one (27.7%) patients didn't return to any sport, with 19 (17.0%) still had planned to return, and 12 (10.7%) gave up sport. The ACL-RSI (Cn) demonstrated excellent validity with all hypotheses confirmed. The outcome of ACL-RSI (Cn) was strongly correlated the KOOS subscale quality of life (r = 0.66, p<0.001), the TSK (r = -0.678, p<0.001), the Tegner score (r = 0.695, p<0.001). There was statistic difference between cases returned (68.6 ± 10.1) and didn't return to sport (41.3 ± 17.7), p<0.001; between cases returned to competitive (71.1 ± 8.9) and recreational sport (62.9 ± 10.5), (P = 0.002); between cases who planned to return (50.7 ± 14.1) and gave up sport (26.5 ± 11.7), (P<0.001). The internal consistency (Cronbach's α = 0.96) and test-retest reliability [intra-class correlation coefficient (ICC) = 0.90] was excellent. The measurement error, floor and ceiling effect was satisfactory. Administration time was 3.2 minutes, and no item was missed.

**Conclusions:**

The ACL-RSI (Cn) scale was confirmed as a valid, reliable, and feasible tool for evaluating psychological factors influencing return to sport.

## Introduction

An anterior cruciate ligament (ACL) deficient knee always precludes active sports patients from strenuous activities, especially involving cutting and pivoting [1–6]. After the development of nearly one century, the ACL reconstruction is now routinely performed to restore knee stability with an ultimate goal to bring patients back to sport, in the best condition at their preinjury levels [7, 8].

In literature, the return to sport after ACL reconstruction was satisfactory for professional athletes playing basketball, American football, soccer and hockey, ranging from nearly 80% to over 95% [9–14]. While for general sport active population, the outcome was lower than expected. It was reported that two third patients had not returned to their preinjury levels by 12 months postoperative [15]. In further investigation, the researchers found that the overall rate of return to preinjury level sport was only 60% by two years after surgery [16]. In a systematic review and meta-analysis including 69 articles reporting on over 7500 participates, only 65% patients returned to the preinjury level at a mean of 40 months postoperative [17].

Traditionally, the physical condition was highly emphasised in postoperative rehabilitation [18–27]. Knee stability, muscle strength, and range of motion were usually used to decide whether they could resume sport or not. Interestingly, the knee function after rehabilitation was often good to excellent in most cases by current measurement, while the return to sport is sometimes disappointing. For the seemingly perplexing phenomenon, some researchers suggested that patients might be not well psychologically prepared to return to sport despite physically rehabilitated [28, 29]. Fear of reinjury was proved as a hindrance for returning to sport after ACL reconstruction [29]. In contrast, positive attitude was suggested to be correlated with the higher rate of returning to sport [30, 31].

In 2008, an Australian research group developed a 12-item English questionnaire named the ACL Return to Sport after Injury scale (ACL-RSI), which consisted of three psychological aspects: athletes’ emotions, performance confidence, and risk appraisal [32]. Soon afterwards, the ACL-RSI was proved of efficacy in predicting the return to sport after ACL reconstruction. Now, the tool has been translated and cross-cultural adapted into several languages including Swedish, French, Dutch and Turkish. All versions demonstrated excellent reliability and validation [33–36].

In this diagnostic study, we tried to translate, cross-cultural adapt and validate the ACL-RSI into simplified Chinese.

## Methods and materials

The study was approved by the Huashan Institutional Review Board (HIRB), and verbal consent was gained from participants.

### Translation and cross-cultural adaptation procedure

In this diagnostic study, the ACL-RSI was translated and culturally adapted from English into simplified Chinese by one orthopaedic doctor, one sports medicine surgeon and one physical therapist after obtaining the permission from original author. All translators speak Chinese as their mother language and have good knowledge of both English and Chinese. After discussion on wording and phrasing with a college English teacher, the original version of ACL-RSI (Cn) was confirmed. Then, the translated scale was retranslated into English by two independent native English speakers who know Mandarin Chinese well and have no idea of the original scale.

In the next, a seven-investigator study team were formed to evaluate the translations and reached a consensus on the pretesting version ACL-RSI (Cn). The study group consisted of two senior sports medicine professors, one attending sports medicine doctor, one undergraduate student majoring in sports medicine rehabilitation and the three translators. Then, six patients (3 male, 3 female, mean age of 26.8 years) were included in a pretest. The patients were asked if they have any difficulty in answering the questions and making comments or giving some suggestions, by which we can refine the scale if necessary.

For measurement, a 10-centimeter visual analogue scale (VAS) with the graduation of 1 millimetre was used. The patients could score by ticking or dotting on the scale. The outcome was recorded in one hundred mark system.

### Validity

The ACL-RSI (Cn) was validated according to the COSMIN (Consensus-based Standards for the selection of health Measurement Instruments) international guidelines [37]. Patients with ACL reconstruction between Jan 2015 and September 2015 at our institute were enrolled in this study. The inclusion and exclusion criteria were presented in Table 1.

**Table 1:** The Inclusion and exclusion criteria.

Inclusion criteria	Exclusion criteria
16 years or older	Revision surgery
ACL reconstruction between Jan 2015 and Sept 2015	Multi-ligament surgery
Capable of competitive sports preinjury	Pre-surgery history
	Grade III or IV cartilage lesion
	Subtotal or total meniscectomy
	Injury or working compensation

### Questionnaires

Another four questionnaires were applied in the validity testing of the ACL-RSI (Cn).

A purpose-specific questionnaire was firstly fulfilled by the patients, which includes their basic information, time from surgery, and current activity level by the Tegner score [38]. The validated Chinese version 2000 International Knee Documentation Committee (IKDC) subjective score was applied [39]. The 18 items questionnaire consisted of three aspects: symptoms, sports activities, and function. The validated Chinese version Knee Injury and Osteoarthritis Outcome Score (KOOS) was applied [40]. The KOOS consisted of five subscales, including pain, symptoms, activities of daily living (ADL), sport and recreation function (function), and knee-related quality of life (QoL). The Tampa Scale of Kinesiophobia (TSK), a 17-item questionnaire, was designed to measure fear of movement and potential reinjury during physical activities [41]. A four point Likert scale was used in each item with the total score ranging from 17 to 68. The higher score indicates a high level of fear. The scale was also validated into Chinese recently.

### Return to sport

The activity levels were measured by the Tegner activity score. Besides current levels, the participants needed to mark their preinjury level by recalling. If the Tegner scores equalled, we defined that this patient had returned to sport at preinjury level. For patients who didn’t return to sport, they need to answer whether they still planned to return; for patients who returned to sport, they needed to explain what kind of games they could play, competitive or recreational.

### Reliability

The first 60 patients were asked to fulfill the ACL-RSI (Cn) twice with a time interval of one to two weeks. Total score of the two tests was calculated. The test–retest reliability was evaluated by the intra-class correlation coefficient (ICC) using the two-way random model in an entire agreement pattern. The confidence interval was 95%. Reproducibility was considered to be ‘‘excellent” with (ICC > 0.80), ‘‘good” (0.60 < ICC ≤ 0.80), ‘‘fair” (ICC ≤ 0.40), or poor (ICC ≤ 0.20). The measurement error was assessed by the Limits of Agreement using Bland and Altman plot.

### Feasibility

The feasibility was determined by the response rate and fulfilling time of the ACL-RSI (Cn). The fulfilling time was documented in a minute by responders.

### Floor and ceiling effects

Floor or ceiling effects indicate that the scale may fail in measuring extreme values. For each item, the percentage of patients who scored the lowest and the highest outcomes were documented. The descriptive data were also calculated, which included the mean value and standard deviation. Floor or ceiling effects were confirmed if more than 15% of patients had scored at the limits of the scale.

### Internal consistency

The internal consistency was assessed by the Cronbach’s alpha coefficient, which was considered to be “excellent” if α > 0.90. The correlation between every two items and each item to total score were also calculated. Structural validity was tested by factor analysis using principal components method with maximum variance rotation. The analysis could determine whether there was one construct or not, which laid the theoretical foundation to calculate the total score. Eigenvalue > 1 was the extraction criteria.

### Construct validity

Seven hypotheses proposed by the investigators to test the construct validity

1. Patients who returned to sport had higher ACL-RSI (Cn) score than those who didn’t.
2. Patients who returned to competitive sport score had higher ACL-RSI (Cn) score than those back to the recreational sport.
3. Patients who planned to return to sport had higher ACL-RSI (Cn) score than those who didn’t.
4. Patients who scored high on the Tegner score had high ACL-RSI (Cn) score. The Tegner activity score was highly correlated with ACL-RSI (Cn).
5. Patients who scored high on the subjective IKDC had high ACL-RSI (Cn) score. The subscale sports activity was highly correlated with ACL-RSI (Cn).
6. Patients who scored highly on the KOOS had high ACL-RSI (Cn) score. The subscales QoL and function were each highly correlated with ACL-RSI (Cn).
7. Patients who scored high on the TSK had low ACL-RSI (Cn) score. The outcomes of the two scales were in a negative correlation.

The construct validity was tested by the Pearson’s correlation between ACL-RSI and the TSK, the Tegner score, the subscales of the subjective IKDC and the KOOS. The correlation could be considered in five degrees: “very strong” (.80< r <1.0), “strong” (.60< r <0.79), “moderate” (.40< r <0.59), “weak” (.20< r <0.39), “very weak” (.00< r <0.19). Discriminant validity was tested by the first three hypotheses using the Mann–Whitney test. P< 0.05 was considered as statistical significance. The analyses were performed by SPSS 19.0 and Medcalc 15.6.

## Results

### Cross-cultural adaptation

The ACL-RSI (Cn) and retranslated scale did not cause any significant grammar or wording problem. The word “fearful” and “afraid”, respectively from item 7 and 9, were synonyms to some extent in Chinese. To distinguish them, we translated the “fearful” into “rang nin wei ju” and converted “play your sport” into the subject of the interrogative sentence. In this way, item 7 was used to assess how “injuring your knee” impressed patients. In contrast, the afraid in item 9 was translated into “hai pa”, evaluating patients’ inner feeling toward accidental injury. As a good example, one patient could find “playing your sport” fearful after ACL reconstruction, but they didn’t afraid of that.

### Study participants

Under criteria, the study finally included 185 patients. Three patients were unavailable in contact, and 62 patients rejected investigation including three failed cases. A total of 120 patient agreed to join in the study, while 112 patients returned final outcomes. Among those who replied, 82 were male, the mean age is 26.6 ± 7.6 (16 to 48) years, and the mean BMI was 24.2 ± 3.9 (17.9 to 30.1). All patients were capable of competitive sport preinjury. The average follow-up time was 15.6 ± 1.9 (11 to 20) months.

### Return to sport

Eighty-one (72.3%, 81/112) patients returned to sport, in which 57 (50.9%, 57/112) patients returned to the competitive sport, and 24 (21.4%, 24/112) patients returned to the recreational sport. Thirty-one (27.7%, 31/112) patients didn’t return to any kind sport. Among them, 19 (17.0%, 19/112) patients still had the plan to return, while 12 (10.7%, 12/112) patients no longer wanted to return to sport. Fifty-one (45.5%, 51/112) patients returned to sport at preinjury level.

### Internal consistency

The principal component analysis demonstrated the unidimensionality with an eigenvalue of 8.3. The sole factor explained 68.8% of the whole content. The internal consistency of the ACL-RSI (Cn) was excellent with a Cronbach’s α of 0.96, which demonstrated very strong correlation among the 12 items. The inter-item correlations were strong, with a mean value of 0.64 (from 0.59 to 0.71). The correlations between each item and the total score were very strong, with a mean value of 0.83 (from 0.65 to 0.89).

### Construct validity

All seven hypotheses were confirmed. The ACL-RSI (Cn) demonstrated significant correlation with all reference tools except the KOOS-ADL. The correlations were weak with the subscales including IKDC symptom and KOOS-symptom. The Strong negative correlation was observed with the TSK. Strong positive correlation was found with the KOOS-QoL. The strongest negative correlation was found with the Tegner score. Strong positive correlation was also observed with the KOOS-QoL. (Table 2)

**Table 2:** Mean values of scales and correlations between the ACL-RSI (Cn) scale and other scales.

Scale	Mean Score (SD)	Correlation with ACL-RSI(Cn)	P-value
ACL-RSI (Cn)	61.3 ± 17.8	-	-
IKDC-symptom	31.6 ± 3.9	r = 0.376	p<0.001
IKDC-function	8.5 ± 0.9	r = 0.483	p<0.001
IKDC-sports	38.3 ± 1.7	r = 0.408	p<0.001
KOOS-pain	92.6 ± 6.1	r = 0.463	p<0.001
KOOS-symptom	89.6 ± 8.4	r = 0.265	p = 0.005
KOOS-ADL*	96.5 ± 3.4	r = 0.167	p = 0.078
KOOS-function**	87.3 ± 8.7	r = 0.520	p<0.001
KOOS-QoL#	82.2 ± 10.2	r = 0.660	p<0.001
TSK##	41.3 ± 11.0	r = -0.678	p<0.001
Tegner Score	6.2 ± 2.0	r = 0.695	p<0.001

*Activities of Daily Life

**Sport and Recreation Function

^#^Knee-related Qualify of Life

^##^Tempa Scale of Kinesiophobia

**Fig 1:**
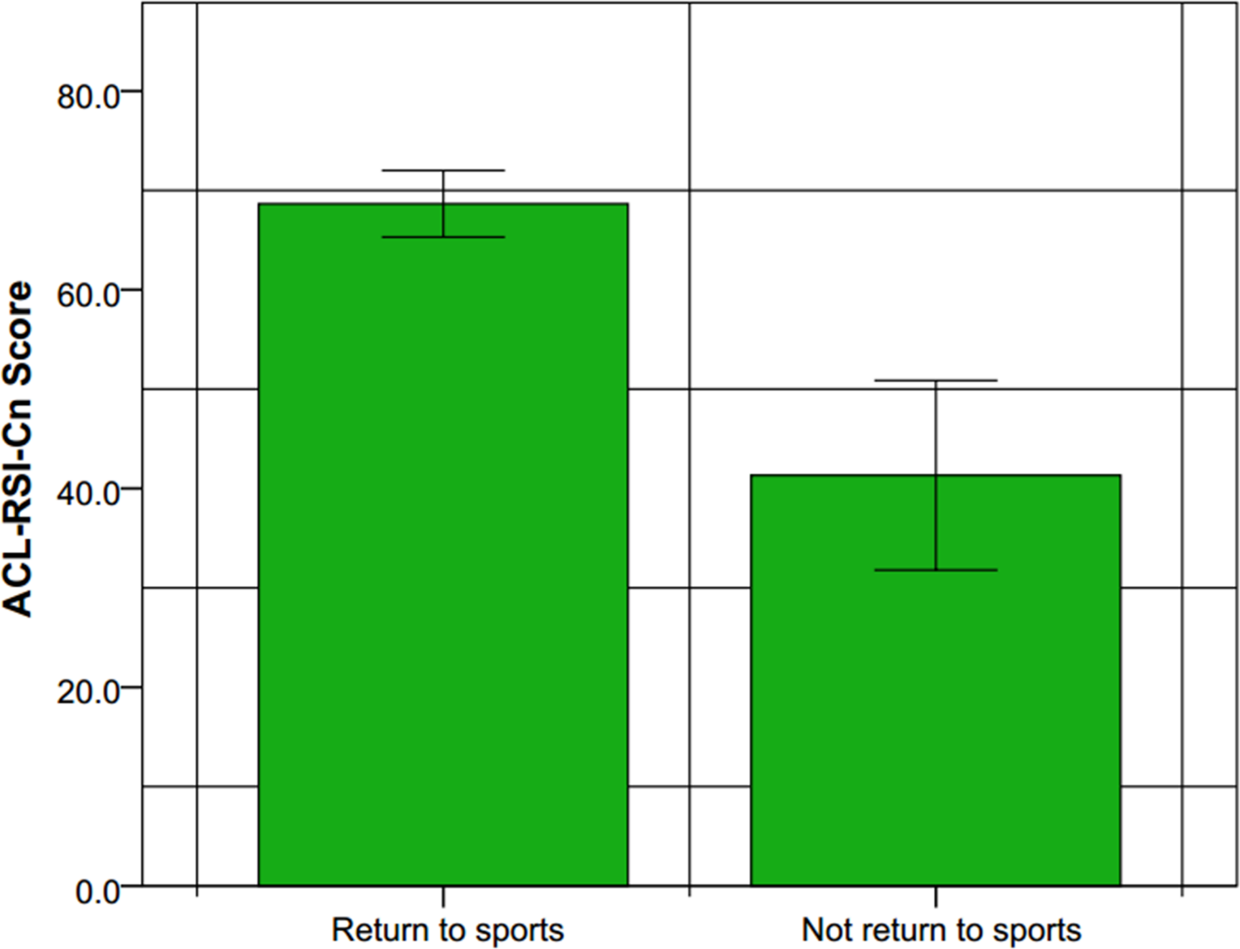
Scores of the ACL-RSI scale at a mean of 15.6 months postoperative, between cases returned to sport and those didn’t return. There was the significant statistical difference, (P<0.001).

**Fig 2:**
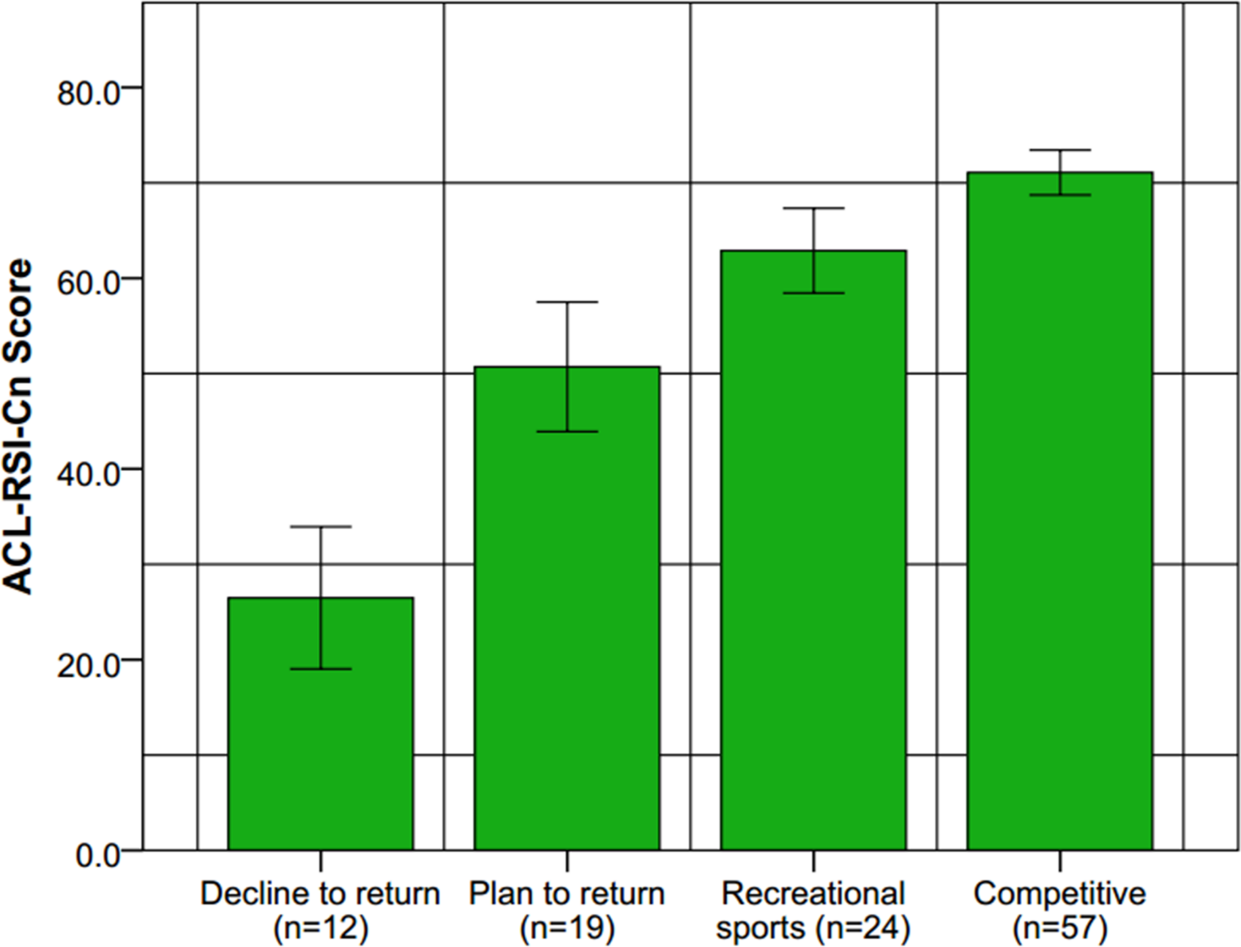
Scores of the ACL-RSI scale at a mean of 15.6 months postoperative of cases returned to the competitive sport, to recreational sport, planned to return, and gave up sport. There was the significant statistical difference between cases returned to the competitive sport and those to recreational sport, (P = 0.002); between cases planned to return to sport and those gave up, (P<0.001).

The discriminant validity was excellent. The score of ACL-RSI (Cn) was respectively 68.6 ± 10.1 (from 45.1 to 87.1) or 41.3 ± 17.7 (from 9.8 to 72.2) in patients who had returned or had not returned to sport, (P<0.001); respectively 71.1 ± 8.9 (from 47.9 to 87.1) or 62.9 ± 10.5 (from 45.1 to 82.3) in patients who had returned to competitive or recreational sport, (P = 0.002); respectively 50.7 ± 14.1 (from 22.9 to 72.2) or 26.5 ± 11.7 (from 9.8 to 45.2) in patients who planned to return or gave up sport, (P<0.001). (Figs 1 and 2)

### Reliability

**Fig 3:**
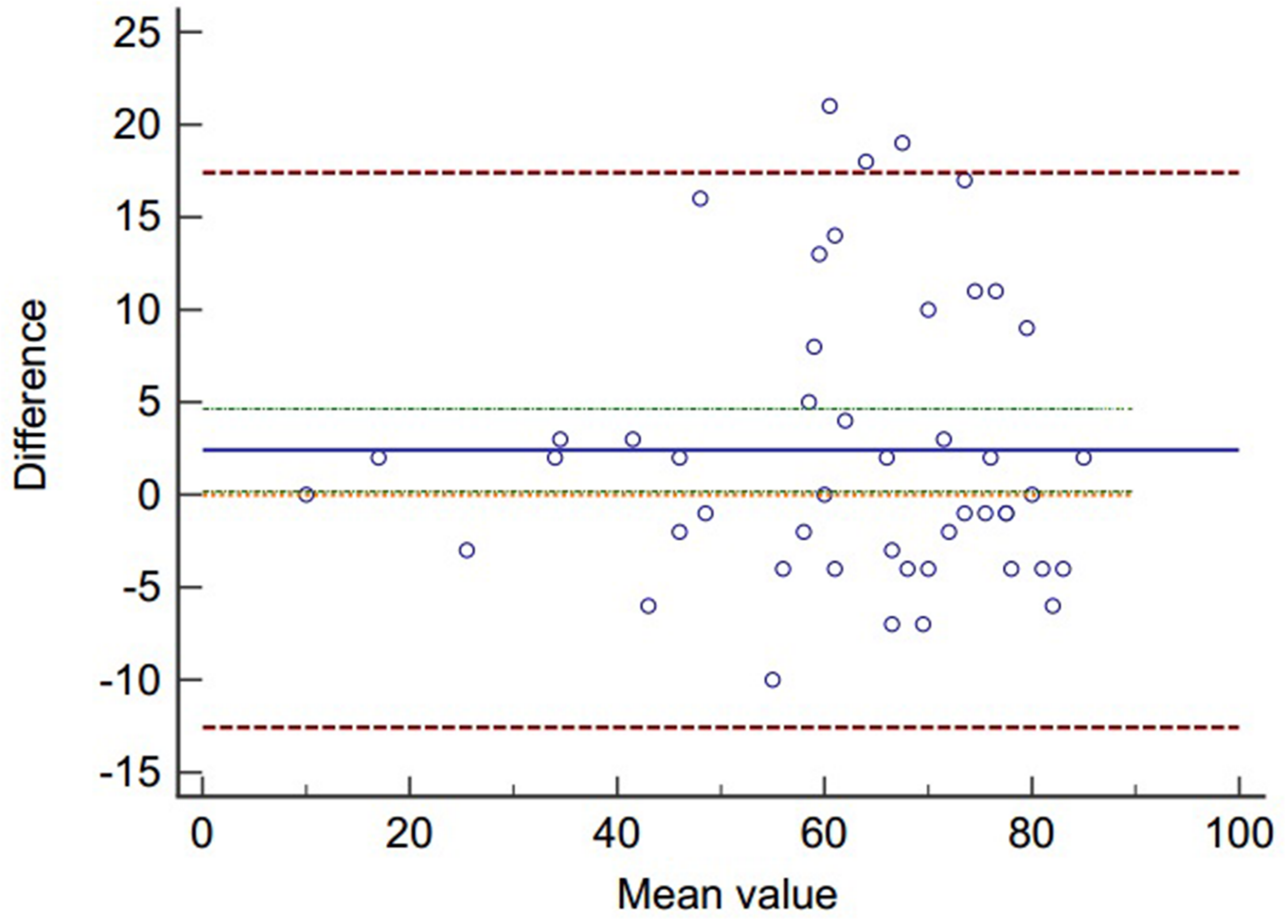
Bland and Altman's diagram are showing good agreement between the two measurements.

The test–retest reliability of the ACL-RSI (Cn) was excellent. Forty-eight patients again answered the ACL-RSI (Cn). The mean total score of the ACL-RSI at first test was 63.1 ± 17.8 and 60.6 ± 17.6 at the second test. The intra-class correlation coefficient (ICC) was 0.90 (from 0.82 to 0.94). The measurement error was satisfactory with Bland and Altman's plot showing good agreement. (Fig 3).

### The floor and ceiling effect

Floor and ceiling effects for each item were acceptable. For floor effect, the percentage of score zero for each question ranged from 0 to 6.3%. For ceiling effect, the percentage of 100 scores for each question ranged from 0 to 5.4%. The overall mean score was 63.1 ± 17.8, which was also satisfactory.

### Feasibility

No item of the ACL-RSI (Cn) was missed. The average fulfilling time of the questionnaire was 3.2 minutes.

## Discussion

The desire of a return to sport is always the impetus of ACL reconstruction for most patients. And such desire also influences doctors’ decisions on patient selection. Albeit the knee function in most patients has been physically recovered, the return to sport rate was not ideal from literature [16, 17, 42, 43]. One explanation was that the lower rate of return to sport might be due to the inferior psychological preparation of patients. Fear of reinjury, negative emotion, shifts in priority, and individual personalities have been reported in association with unsatisfactory return to sport [28–30]. Specifically, fear of injury has been proved as a definite hindrance by several investigators using different methods [29].

In previous research of psychological factors related to return to sport after the ACL injury, several tools have been applied, including the Knee-Self-Efficacy Scale (K-SES) [44], the Emotional Responses of Athletes to Injury Questionnaire (ERAIQ) [45, 46], the Tampa Scale of Kinesiophobia (TSK) [47], and an Italian psychovitality scale [48]. While these tools were not developed for patients with ACL reconstruction. The ACL-RSI, a 12-item scale measuring emotions, confidence in performance and risk appraisal of athletes, was specially designed by Webster et al. to evaluate psychological impacts on returning to sport after ACL reconstruction. It has been proved with good efficacy in following studies. And the scale also has been successfully translated into several languages.

During our clinical work, it has long been noticed that quite a few patients somehow have failed to return to sport, especially at their preinjury level despite good to excellent knee function. By interview conversation, we found that some patients were afraid of reinjury and the whole process of surgery and postoperative rehabilitation was fearful to them. Also, the shift of priority made them more rational than emotional in participating fierce body contact games. Some patients expressed that they were no longer confident or passionate enough on the same field and decide to modify their sport activities to a lower intensity, i.e. from competitive to recreational sport.

Traditional measurement tools used in follow-up after ACL reconstruction mainly focused on the evaluation of clinical symptoms and function of the affected limb (the KOOS, the IKDC subjective score), as well as the patient's activity level (Tegner Activity Score). While their measuring spectrum failed to include the patients’ psychological conditions. Recently, it was reported that the returning to sports should be delayed at nearly two years postoperative to reduce the incidence of second ACL injuries. For the incidence rate in high-level athletes within two years postoperative, it might be due to the insufficient biological and functional recovery of the affected knee as the researchers suggested, while psychological factors could also be contributive to returning to sports from our academic point.

For quantitative investigation of this issue, an efficient tool for psychological measurement is needed. And it will be a great help to doctors, physical therapists, and the patients of ACL reconstruction, who can eventually get safe and predictive clearance to return to sport when they are fully prepared, both physically and psychologically. Therefore we decided to translate the ACL-RSI into simplified Chinese. The simplified Chinese version of the ACL-RSI was proved to be a reliable, valid and feasible questionnaire to assess psychological factors of the patients after ACL reconstruction.

Although the ACL-RSI (Cn) consisted of three psychological aspects including emotion, confidence in the performance and risk appraisal, the factor analysis indicated that it was still unidimensional. In other words, the three psychological factors cannot be separated. Also, the high inter-item correlation also revealed its strong internal consistency.

Four questionnaires were used in the validation. The simplified Chinese version IKDC was a validated tool widely applied by Chinese sport medicine doctors and orthopaedic surgeons. The Tegner activity score, a simple but useful tool, was routinely used in China to evaluate the activity level of patients. The simplified Chinese version KOOS was validated in 2006. As a disease-specific instrument for knee OA, the KOOS was developed from the Western Ontario and McMaster Universities Osteoarthritis Index (WOMAC) and was designed for younger and more active patients. All questionnaires were applied in other studies completed or still ongoing in our institute. The simplified Chinese version TSK was validated in patients with low back pain after cultural adaptation. Kinesiophobia means the fear of movement or (re)injury, which has been proved as a hindrance of return to sport after ACL reconstruction. Therefore, we used the simplified Chinese TSK to test the construct validation of ACL-RSI. In this study, the correlation between ACL-RSI and the KOOS subscale QoL was strong, which was also found in the Swedish version, French version. In the Turkish version, the correlation between ACL-RSI and KOOS-QoL was the highest one. From our perspective, this is due to the intrinsic property of KOOS-QoL, which involves risk appraisal management and some psychological factors like confidence and self-awareness. The strongest correlation was observed with the Tegner score, which solidly confirmed the psychological impact on sport activity level.

The ACL-RSI showed excellent discriminant validity, (Fig 1). A significant difference was found between the patients who had returned to sport and those who hadn’t. The outcomes of patients who returned to the competitive sport were higher than those only took part in a recreational sport usually like jogging or cycling. Among the patients who didn’t return to sport, we separated those who still had the plan to return and those who gave up sport, at least on the investigating moment. A significant difference was also found between the two groups.

The reliability of the ACL-RSI scale has been reported of satisfactory outcomes in previous studies. The test-retest reliability of simplified Chinese version ACL-RSI was high. The good agreement shown by the Bland and Altman plot indicated the measurement error is satisfactory.

One limitation of this study is the participating rate. Only 61% of the included patients agreed to participate and eventually returned available data. For the test-retest reliability, 60 patients were primarily supposed to fulfill the ACL-RSI twice, while only 48 patients (80%, 48/60) returned the outcome. One explanation might be that the patients have found tedious in fulfilling the questionnaires. Some of them participated in another study, in which they had already finished some paperwork. Frankly, patients needed to answer at maximum over one hundred questions in this study. In a self-voluntary rather than mandatory or financial rewarded mode, we have been much appreciated that.

The included patients were all capable of the competitive sport before the injury. This sample selection might be criticised that it could not represent all patients with ACL reconstruction, while the ACL reconstruction should have been a surgery for patients who need high-level sport activity.

## Conclusion

The simplified Chinese version ACL-RSI scale was confirmed as a valid, reliable, and feasible tool for evaluating psychological factors influencing return to sport. This tool may help Chinese ACL reconstructed population both psychologically and physically ready to return to sport.

## Supporting information

S1 Appendix Simplified Chinese version of the Anterior Cruciate Ligament Return to Sport after Injury (ACL-RSI) scale. (PDF)

S2 Appendix The Anterior Cruciate Ligament Return to Sport after Injury (ACL-RSI) scale-Original Version. (PDF)

S3 Appendix The Underlying participant-level data. (PDF)
