# Supplementary material for: Translation, cultural adaptation and validation of simplified Chinese version of the anterior cruciate ligament return to sport after injury (ACL-RSI) scale
Source: PLoS One. 2017 Aug 17;12(8):e0183095. doi: 10.1371/journal.pone.0183095 (PMC5560729; doi:10.1371/journal.pone.0183095)

前交叉韧带伤后重返运动量表

? 姓名：____________________

性别：____________________

年龄：____________________

身高：____________________

体重：____________________

伤侧：____________________

受伤时间：____________________

手术时间：____________________

是否合并其他损伤（如有，请明确）：____________________

是否重返运动（如重返，请明确时间）：____________________

是否重返术前运动水平（如重返，请明确时间）：____________________

联系方式：____________________

电话：____________________

电子邮箱：____________________

填表日期：____________________

- 量表包含 12 项问题，请填表人根据实际情况在 10cm 线上作出选择。
- “10”、“0”分别代表最大、最小程度。
- 请您根据自己的实际感受做出评价，任一刻度均可勾选。

1. 您有信心在体育活动中恢复到伤前运动水准么？

0 1 2 3 4 5 6 7 8 9 10

2. 您认为自己很可能在参加运动时再次损伤膝关节么？

0 1 2 3 4 5 6 7 8 9 10

3. 您在参加体育活动时会感到紧张么？

0 1 2 3 4 5 6 7 8 9 10

4. 您有信心在参加运动时不发生膝关节打软么？

0 1 2 3 4 5 6 7 8 9 10

5. 您有信心在参加运动时不为自己的膝关节感到担忧么？

0 1 2 3 4 5 6 7 8 9 10

6. 您会因必须在运动中顾及自己的膝关节而感到沮丧么？

0 1 2 3 4 5 6 7 8 9 10

7. 参加运动所致的膝关节再次损伤让您担心么？

0 1 2 3 4 5 6 7 8 9 10

8. 您对自己的膝关节顶住压力有信心么？

0 1 2 3 4 5 6 7 8 9 10

9. 您害怕自己在参加运动时遭受膝关节的意外损伤么？

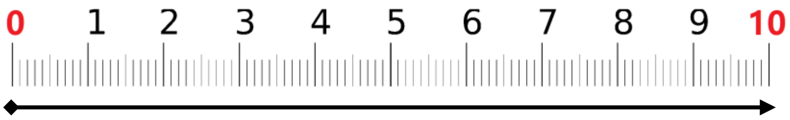

10. 损伤后必须再次经历手术及康复的想法会阻碍您参加运动么？

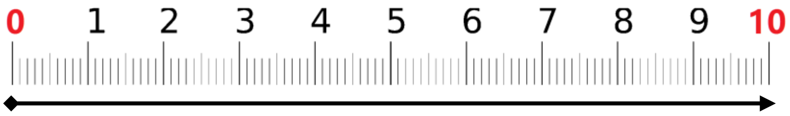

11. 您对自己在参加运动时具备良好表现的能力有信心么？

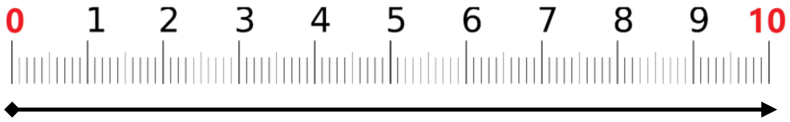

12. 您在参加运动时感到轻松么？

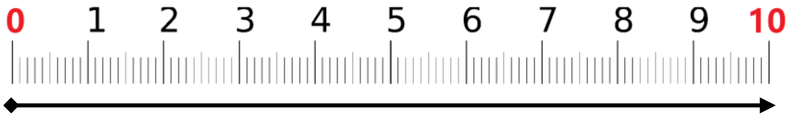

Supplement: S1 Appendix — (PDF) [file pone.0183095.s001.pdf]
