# Supplementary material for: Translation, cultural adaptation and validation of simplified Chinese version of the anterior cruciate ligament return to sport after injury (ACL-RSI) scale
Source: PLoS One. 2017 Aug 17;12(8):e0183095. doi: 10.1371/journal.pone.0183095 (PMC5560729; doi:10.1371/journal.pone.0183095)
Supplement: S2 Appendix — (PDF) [file pone.0183095.s002.pdf]

Scale item	Order in scale
Emotions	
1. Are you nervous about playing your sport?	3
2. Do you find it frustrating to have to consider your knee with respect to your sport?[a]	6
3. Do you feel relaxed about playing your sport?	12
4. Are you fearful of re-injuring your knee by playing your sport?	7
5. Are you afraid of accidentally injuring your knee by playing your sport?	9
Confidence in performance	
6. Are you confident that your knee will not give way by playing your sport?	4
7. Are you confident that you could play your sport without concern for your knee?	5
8. Are your confident about your knee holding up under pressure?	8
9. Are you confident that you can perform at your previous level of sport participation?	1
10. Are you confident about your ability to perform well at your sport?	11
Risk appraisal	
11. Do you think you are likely to re-injure your knee by participating in your sport?	2
12. Do thoughts of having to go through surgery and rehabilitation again prevent you from playing your sport?	10
