# Supplementary material for: Translation, cultural adaptation and validation of simplified Chinese version of the anterior cruciate ligament return to sport after injury (ACL-RSI) scale
Source: PLoS One. 2017 Aug 17;12(8):e0183095. doi: 10.1371/journal.pone.0183095 (PMC5560729; doi:10.1371/journal.pone.0183095)
Supplement: S3 Appendix — (PDF) [file pone.0183095.s003.pdf]

Participant	Age	Gender	BMI	Fu	ACL-RSI	RSI1	RSI2
Case 1	32	1	22.1	17	67	55	73
Case 10	34	2	22.4	12	73	60	82
Case 100	18	1	21.5	15	23	30	20
Case 101	19	2	25.7	18	64	71	82
Case 102	20	1	28.8	15	66	75	63
Case 103	18	2	28.7	18	79	85	80
Case 104	30	1	29.1	16	74	80	85
Case 105	29	1	18.6	18	62	55	58
Case 106	30	1	29.8	17	66	95	60
Case 107	20	1	25	17	70	80	55
Case 108	35	1	18.2	18	25	50	10
Case 109	22	1	23.3	17	37	45	38
Case 11	41	1	21.2	18	75	90	78
Case 110	29	1	26.3	15	59	70	45
Case 111	35	1	19.7	17	45	61	52
Case 112	18	1	19.2	13	73	60	85
Case 12	36	1	26.7	14	66	73	65
Case 13	26	1	19	18	40	60	40
Case 14	18	2	19.1	15	48	75	30
Case 15	28	2	20.4	12	60	70	56
Case 16	19	2	27.3	17	77	85	85
Case 17	30	2	22	15	75	88	55
Case 18	19	1	21.6	15	71	80	65
Case 19	32	2	27.6	14	79	90	80
Case 2	23	2	29.1	16	52	40	35
Case 20	34	2	18.9	12	63	55	60
Case 21	23	2	29.5	13	68	50	70
Case 22	34	2	27.5	12	66	100	52
Case 23	30	1	17.9	14	68	85	75
Case 24	30	2	19.6	15	18	20	22
Case 25	17	2	21.5	16	66	65	55
Case 26	39	1	21.1	16	82	95	95
Case 27	29	2	20	12	54	70	50
Case 28	18	2	20.4	15	86	90	95
Case 29	33	2	20.6	18	61	75	65
Case 3	21	1	19.5	16	68	78	54
Case 30	26	2	21.1	16	66	50	44
Case 31	20	1	28.6	15	57	75	50
Case 32	31	1	23.5	18	80	90	85
Case 33	35	2	28.7	14	53	60	61
Case 34	33	1	29.1	17	71	80	70
Case 35	21	1	27.1	17	57	70	50
Case 36	18	1	20.3	15	43	55	40
Case 37	29	1	24	14	47	55	30
Case 38	20	1	27.5	15	64	75	65

Case 39	21	1	26	18	76	95	75
Case 4	17	1	21.5	14	77	100	90
Case 40	18	1	20.2	14	73	60	50
Case 41	23	2	30.1	15	76	85	75
Case 42	20	1	24.2	18	24	25	40
Case 43	20	1	24.5	13	59	80	55
Case 44	26	1	26.4	18	65	75	60
Case 45	27	1	20.6	20	35	25	65
Case 46	43	1	27.1	19	82	95	60
Case 47	18	1	18	14	45	70	54
Case 48	31	1	20.2	15	84	90	85
Case 49	20	1	20.6	15	68	74	65
Case 5	31	1	27.8	15	57	70	60
Case 50	21	1	28.6	15	37	44	32
Case 51	24	1	22.1	15	62	71	60
Case 52	35	2	27.8	16	50	45	35
Case 53	32	1	27.8	16	62	75	65
Case 54	20	1	19.9	13	30	40	30
Case 55	48	1	27.8	16	48	35	40
Case 56	16	1	26.4	15	63	71	60
Case 57	28	1	28.7	19	80	64	75
Case 58	47	1	26.2	17	72	80	70
Case 59	28	1	23.3	18	86	94	95
Case 6	31	2	21.9	15	66	75	65
Case 60	31	1	25	16	45	40	55
Case 61	17	2	20.9	16	79	75	75
Case 62	25	1	18.3	19	80	60	92
Case 63	23	1	28.6	17	87	100	90
Case 64	39	1	30	13	64	75	65
Case 65	19	1	19.1	18	10	20	5
Case 66	20	1	29.7	15	76	75	80
Case 67	27	1	25.7	12	71	90	80
Case 68	24	2	21.6	14	70	87	75
Case 69	27	1	25.8	17	48	63	45
Case 7	40	2	20.5	17	77	80	83
Case 70	36	1	29	15	54	65	58
Case 71	28	1	25.6	14	67	79	70
Case 72	21	1	27.5	15	36	57	40
Case 73	46	1	29.4	16	67	77	70
Case 74	23	1	24	17	25	35	25
Case 75	30	1	28.9	18	62	80	40
Case 76	31	1	28.1	16	69	60	50
Case 77	22	2	29	14	13	18	10
Case 78	33	1	18.1	15	81	83	82
Case 79	38	1	25.9	11	10	10	5
Case 8	21	2	20.4	15	36	45	30

Case 80	29	1	26.3	16	81	80	75
Case 81	21	1	18.7	14	71	45	88
Case 82	33	1	24.4	15	68	82	75
Case 83	27	2	18.4	14	56	54	57
Case 84	22	1	27.2	16	62	80	70
Case 85	44	1	29.3	16	45	55	45
Case 86	19	1	26.6	16	79	95	85
Case 87	25	1	29.5	14	60	75	60
Case 88	26	1	27.9	18	84	100	90
Case 89	20	1	28.1	14	72	82	70
Case 9	17	1	20.7	16	63	15	55
Case 90	33	1	22.6	18	57	60	65
Case 91	20	1	28.5	13	60	72	60
Case 92	17	1	18.4	15	64	70	65
Case 93	20	1	29.7	17	73	88	75
Case 94	19	1	27.5	16	77	95	85
Case 95	37	2	18.1	12	59	60	68
Case 96	22	2	19.2	16	71	55	75
Case 97	22	1	24.7	16	66	75	65
Case 98	16	1	26.1	18	35	40	49
Case 99	21	1	18.8	15	77	95	85

RSI3	RSI4	RSI5	RSI6	RSI7	RSI8	RSI9	RSI10
72	75	80	60	65	65	60	62
80	75	80	60	80	87	60	70
15	22	30	10	0	15	10	23
55	65	70	45	40	71	50	72
61	56	75	51	65	75	57	65
75	65	85	90	60	90	65	85
85	80	65	80	57	89	60	80
55	77	70	75	40	70	50	71
22	65	65	60	40	70	81	100
65	75	70	85	50	80	60	80
15	5	5	10	0	20	20	50
25	30	33	61	15	46	15	40
80	75	20	85	95	85	81	79
30	65	70	75	40	70	51	70
21	24	0	73	32	60	45	62
80	95	53	62	70	95	62	80
60	73	71	50	65	75	52	65
32	60	40	25	20	35	22	40
35	45	40	30	65	65	20	50
55	63	55	50	65	66	40	60
90	70	70	75	80	90	60	84
70	80	80	65	80	88	65	75
60	80	70	45	70	78	70	75
75	70	85	75	75	85	60	78
75	69	42	60	20	68	60	55
64	50	60	60	75	80	50	70
80	70	70	88	70	75	55	60
48	65	65	40	55	65	73	91
70	80	65	65	75	65	50	60
20	50	20	10	0	25	0	10
65	61	70	65	75	74	50	70
92	70	95	75	75	65	70	100
42	60	65	44	30	62	45	55
90	70	95	85	75	90	70	95
50	70	75	60	45	33	50	70
90	65	85	65	60	90	20	70
95	45	90	78	50	55	70	90
60	70	70	35	45	35	45	70
83	70	80	85	80	70	70	85
55	50	50	30	30	60	60	55
65	75	80	65	80	80	56	80
45	53	52	38	42	65	48	81
35	45	40	30	60	35	25	50
40	50	51	40	60	55	30	55
60	70	70	60	45	70	50	70

42	80	81	60	55	85	75	90
100	80	70	70	5	95	70	97
80	55	95	76	70	75	75	95
82	100	80	90	55	85	60	80
35	40	40	25	20	15	15	25
30	65	65	45	40	70	60	77
60	66	72	66	60	77	50	70
35	72	37	30	15	50	10	20
82	100	75	83	80	93	70	85
35	55	40	30	60	50	15	45
80	90	90	85	85	95	50	90
62	68	75	70	66	73	51	72
55	50	60	52	40	55	55	70
22	42	45	60	10	45	15	40
58	65	65	46	66	75	45	62
50	40	60	49	60	65	40	60
72	50	70	42	60	65	50	65
25	35	31	15	10	40	12	43
35	45	40	25	75	70	30	100
75	65	60	40	65	75	50	65
80	85	75	70	75	90	87	85
70	75	75	56	55	80	60	90
100	100	80	85	90	85	82	60
55	65	70	55	70	75	50	70
50	51	60	40	40	35	31	40
80	81	90	73	80	90	65	75
81	94	92	73	81	76	66	90
85	95	75	75	85	95	65	100
68	54	70	50	45	77	50	70
12	12	5	0	0	5	10	20
85	60	75	80	70	65	70	70
80	75	80	65	60	80	60	60
82	75	80	55	55	80	55	55
30	55	44	30	25	55	40	60
60	95	80	90	100	85	95	65
52	60	55	42	35	61	40	60
65	75	70	55	32	80	50	72
42	20	40	25	10	25	33	50
55	70	80	40	80	60	50	70
12	45	30	14	0	15	10	30
35	70	75	55	50	80	30	77
66	78	80	60	55	85	66	80
5	16	15	10	0	5	10	20
76	80	90	70	65	85	70	95
0	10	10	0	0	35	10	10
22	65	41	10	4	45	0	100

70	81	90	95	80	88	99	90
80	95	85	66	65	90	40	55
75	80	81	60	59	85	30	35
70	65	51	68	30	50	50	50
65	76	75	55	50	80	20	20
40	50	50	30	25	55	30	50
90	85	92	70	65	95	40	70
55	80	65	45	40	71	30	70
86	95	80	72	70	95	35	92
65	75	88	50	50	80	61	80
73	78	75	55	70	70	60	83
45	44	60	70	30	30	70	100
55	80	62	45	40	70	50	60
60	70	70	60	45	75	60	70
81	80	75	75	55	85	65	83
100	70	70	55	61	85	75	90
50	70	65	55	40	55	50	70
75	80	73	75	55	75	63	80
65	72	70	65	45	75	55	73
25	60	35	45	10	40	20	30
80	93	74	75	60	85	75	90

RSI11	RSI12	Pain	Symptom	ADL	Function
85	50	94.4	96.4	100	90
90	56	91.7	89.3	94.1	75
40	60	94.4	96.4	100	85
75	70	91.7	78.6	91.2	90
82	65	94.4	96.4	94.1	95
90	75	97.2	92.9	94.1	95
85	40	97.2	96.4	100	90
75	50	80.5	75	97.1	90
95	40	97.2	96.4	94.1	75
85	60	94.4	89.3	97.1	95
62	50	80.5	78.6	98.5	70
50	40	80.5	71.4	88.2	75
82	55	97.2	96.4	100	100
75	46	100	96.4	100	90
61	50	80.5	75	86.8	100
90	40	80.5	85.7	95.6	95
75	70	97.2	92.9	97.1	80
50	56	83.3	85.7	97.1	85
50	70	83.3	78.6	91.2	75
70	65	86.1	89.3	97.1	95
90	40	100	92.9	97.1	95
75	80	97.2	92.9	97.1	100
85	76	100	100	100	95
90	85	86.1	75	97.1	80
65	35	94.4	85.7	98.5	95
80	50	91.7	89.3	95.6	100
85	45	97.2	92.9	97.1	95
100	40	97.2	96.4	100	100
50	70	97.2	92.9	97.1	85
25	12	88.9	89.3	95.6	75
80	61	86.1	71.4	91.2	80
60	95	100	100	100	100
65	62	97.2	85.7	94.1	80
80	95	100	100	100	95
70	70	91.7	92.9	100	80
70	70	94.4	96.4	97.1	80
80	50	86.1	75	98.5	70
70	60	97.2	92.9	97.1	80
90	75	97.2	96.4	94.1	95
65	55	97.2	92.9	94.1	85
80	40	100	96.4	100	95
70	65	88.9	82.1	98.5	70
50	46	91.7	92.9	97.1	85
50	50	91.7	92.9	98.5	90
60	70	97.2	85.7	94.1	80

95	81	80.5	71.4	91.2	85
98	44	97.2	96.4	100	95
85	57	97.2	96.4	100	80
82	40	86.1	78.6	95.6	100
7	5	88.9	89.3	97.1	85
77	40	94.4	96.4	100	95
70	50	94.4	96.4	100	100
30	25	88.9	71.4	95.6	85
95	63	83.3	75	95.6	90
50	38	91.7	89.3	97.1	85
80	91	100	96.4	100	100
67	70	94.4	92.9	100	90
70	50	97.2	92.9	97.1	80
43	40	86.1	78.6	94.1	85
70	65	91.7	92.9	95.6	90
60	40	97.2	96.4	100	90
78	50	97.2	85.7	97.1	85
40	35	88.9	89.3	95.6	75
50	30	91.7	92.9	97.1	90
70	65	97.2	71.4	95.6	95
81	95	100	96.4	100	90
80	75	94.4	96.4	97.1	85
100	60	100	100	100	100
65	72	97.2	96.4	100	85
40	60	91.7	92.9	97.1	80
78	90	97.2	96.4	100	85
91	60	100	100	100	95
100	80	100	89.3	95.6	100
70	70	100	100	100	95
15	13	83.3	96.4	100	75
86	90	94.4	96.4	97.1	85
70	55	97.2	96.4	100	80
70	65	97.2	92.9	97.1	80
65	60	91.7	96.4	100	70
50	36	94.4	96.4	97.1	90
61	60	94.4	89.3	98.5	95
75	75	80.5	85.7	91.2	90
45	40	88.9	89.3	94.1	85
80	75	88.9	89.3	94.1	95
40	45	80.5	78.6	95.6	70
85	65	94.4	89.3	97.1	90
90	55	88.9	75	88.2	95
30	20	97.2	85.7	91.2	80
100	80	100	92.9	91.2	95
20	10	83.3	75	88.2	70
30	45	88.9	92.9	100	80

70	51	100	92.9	95.6	90
90	50	80.5	71.4	88.2	90
85	70	83.3	75	91.2	75
80	45	91.7	89.3	97.1	80
80	70	91.7	100	100	80
55	60	91.7	92.9	94.1	70
96	70	94.4	85.7	89.7	95
70	60	97.2	96.4	100	80
100	92	100	92.9	89.7	95
80	80	100	100	100	100
85	38	97.2	96.4	95.6	100
60	50	88.9	89.3	97.1	85
70	50	83.3	78.6	97.1	90
75	50	86.1	71.4	97.1	80
65	45	97.2	96.4	94.1	95
95	40	100	100	100	80
75	53	94.4	96.4	100	100
90	60	91.7	96.4	100	85
71	65	94.4	85.7	98.5	80
40	20	80.5	78.6	88.2	85
60	50	91.7	92.9	100	95

QoL	TSK	RTS	IKDC Function 87	IKDC Sports 87	IKDC Symptom 87
87.5	36	2	9	39	36
93.8	42	2	7	40	30
81.3	57	3	8	36	36
93.8	49	2	9	40	30
81.3	53	1	9	36	36
81.3	40	2	9	38	35
75	36	2	9	38	36
93.8	48	2	9	39	25
87.5	43	3	7	33	35
87.5	27	1	9	37	30
75	57	4	7	37	26
56.3	36	4	7	36	27
93.8	33	2	10	38	34
75	50	1	9	36	37
62.5	38	1	10	40	29
93.8	36	2	9	38	29
75	39	1	8	39	36
75	34	3	9	36	28
75	49	1	8	38	26
87.5	42	2	9	40	29
93.8	50	2	9	36	33
75	40	2	10	40	35
93.8	43	2	9	39	37
81.3	37	1	8	39	28
81.3	59	1	9	36	31
75	34	1	10	37	26
81.3	34	2	9	39	35
87.5	49	3	10	40	32
93.8	35	1	7	38	33
75	50	4	8	40	28
75	40	1	10	40	23
81.3	22	1	10	40	35
75	45	3	8	35	30
93.8	19	2	9	40	37
93.8	64	3	8	38	27
75	32	1	8	38	31
75	34	1	7	37	25
62.5	54	3	8	39	36
93.8	31	2	9	40	36
75	50	2	8	40	36
81.3	36	2	9	38	36
81.3	46	3	7	37	30
93.8	51	3	8	40	30
75	46	1	9	38	30
75	36	1	8	38	27

75	30	2	8	39	29
93.8	40	2	9	40	36
81.3	24	1	8	39	34
93.8	20	2	10	40	26
68.8	50	4	8	34	28
81.3	40	2	9	38	32
93.8	37	2	10	40	32
62.5	40	4	8	34	26
93.8	51	2	9	40	25
75	43	3	9	39	29
100	20	2	10	40	37
81.3	33	2	9	40	35
75	33	3	8	37	34
87.5	48	3	8	37	30
93.8	51	2	8	39	30
75	50	1	8	37	36
93.8	54	2	8	38	31
62.5	62	4	7	37	30
87.5	52	2	9	40	31
87.5	44	2	9	38	34
93.8	40	2	9	39	36
87.5	33	3	8	37	36
100	29	2	10	40	37
81.3	39	3	8	40	34
68.8	50	4	8	40	32
93.8	34	2	8	39	36
93.8	50	2	9	37	37
93.8	25	2	10	40	34
93.8	37	2	9	38	37
62.5	66	4	7	35	26
75	27	2	8	39	34
81.3	32	2	8	39	33
87.5	36	2	8	40	35
75	60	3	7	35	32
87.5	23	2	9	39	31
68.8	40	2	9	40	33
93.8	30	2	9	40	26
62.5	57	4	8	39	30
93.8	52	2	9	38	27
75	56	3	7	36	26
81.3	40	2	9	40	32
81.3	42	2	9	40	25
68.8	61	4	8	37	30
93.8	26	2	9	39	33
56.3	65	4	7	36	27
75	58	4	8	40	36

93.8	26	2	9	36	33
93.8	41	2	9	38	24
87.5	49	2	7	39	26
81.3	46	3	8	36	32
81.3	41	2	8	40	33
75	44	1	7	38	32
93.8	21	2	9	39	30
75	36	1	8	39	36
93.8	32	2	9	38	35
75	32	2	10	40	37
81.3	35	2	10	39	37
68.8	34	3	8	40	29
75	44	2	9	39	28
81.3	41	1	8	40	26
75	46	2	9	36	32
81.3	35	1	8	40	37
75	23	1	10	40	35
93.8	48	1	8	40	33
93.8	51	2	8	39	32
75	60	3	8	37	26
93.8	24	2	9	38	31

IKDC Final	IKDC Function 100	IKDC Sports 100	IKDC Symptom 100	IKDC Final 100
96.6	10.3	44.8	41.4	96.6
88.5	8.0	46.0	34.5	88.5
92.0	9.2	41.4	41.4	92.0
90.8	10.3	46.0	34.5	90.8
93.1	10.3	41.4	41.4	93.1
94.3	10.3	43.7	40.2	94.3
95.4	10.3	43.7	41.4	95.4
83.9	10.3	44.8	28.7	83.9
86.2	8.0	37.9	40.2	86.2
87.4	10.3	42.5	34.5	87.4
80.5	8.0	42.5	29.9	80.5
80.5	8.0	41.4	31.0	80.5
94.3	11.5	43.7	39.1	94.3
94.3	10.3	41.4	42.5	94.3
90.8	11.5	46.0	33.3	90.8
87.4	10.3	43.7	33.3	87.4
95.4	9.2	44.8	41.4	95.4
83.9	10.3	41.4	32.2	83.9
82.8	9.2	43.7	29.9	82.8
89.7	10.3	46.0	33.3	89.7
89.7	10.3	41.4	37.9	89.7
97.7	11.5	46.0	40.2	97.7
97.7	10.3	44.8	42.5	97.7
86.2	9.2	44.8	32.2	86.2
87.4	10.3	41.4	35.6	87.4
83.9	11.5	42.5	29.9	83.9
95.4	10.3	44.8	40.2	95.4
94.3	11.5	46.0	36.8	94.3
89.7	8.0	43.7	37.9	89.7
87.4	9.2	46.0	32.2	87.4
83.9	11.5	46.0	26.4	83.9
97.7	11.5	46.0	40.2	97.7
83.9	9.2	40.2	34.5	83.9
98.9	10.3	46.0	42.5	98.9
83.9	9.2	43.7	31.0	83.9
88.5	9.2	43.7	35.6	88.5
79.3	8.0	42.5	28.7	79.3
95.4	9.2	44.8	41.4	95.4
97.7	10.3	46.0	41.4	97.7
96.6	9.2	46.0	41.4	96.6
95.4	10.3	43.7	41.4	95.4
85.1	8.0	42.5	34.5	85.1
89.7	9.2	46.0	34.5	89.7
88.5	10.3	43.7	34.5	88.5
83.9	9.2	43.7	31.0	83.9

87.4	9.2	44.8	33.3	87.4
97.7	10.3	46.0	41.4	97.7
93.1	9.2	44.8	39.1	93.1
87.4	11.5	46.0	29.9	87.4
80.5	9.2	39.1	32.2	80.5
90.8	10.3	43.7	36.8	90.8
94.3	11.5	46.0	36.8	94.3
78.2	9.2	39.1	29.9	78.2
85.1	10.3	46.0	28.7	85.1
88.5	10.3	44.8	33.3	88.5
100.0	11.5	46.0	42.5	100.0
96.6	10.3	46.0	40.2	96.6
90.8	9.2	42.5	39.1	90.8
86.2	9.2	42.5	34.5	86.2
88.5	9.2	44.8	34.5	88.5
93.1	9.2	42.5	41.4	93.1
88.5	9.2	43.7	35.6	88.5
85.1	8.0	42.5	34.5	85.1
92.0	10.3	46.0	35.6	92.0
93.1	10.3	43.7	39.1	93.1
96.6	10.3	44.8	41.4	96.6
93.1	9.2	42.5	41.4	93.1
100.0	11.5	46.0	42.5	100.0
94.3	9.2	46.0	39.1	94.3
92.0	9.2	46.0	36.8	92.0
95.4	9.2	44.8	41.4	95.4
95.4	10.3	42.5	42.5	95.4
96.6	11.5	46.0	39.1	96.6
96.6	10.3	43.7	42.5	96.6
78.2	8.0	40.2	29.9	78.2
93.1	9.2	44.8	39.1	93.1
92.0	9.2	44.8	37.9	92.0
95.4	9.2	46.0	40.2	95.4
85.1	8.0	40.2	36.8	85.1
90.8	10.3	44.8	35.6	90.8
94.3	10.3	46.0	37.9	94.3
86.2	10.3	46.0	29.9	86.2
88.5	9.2	44.8	34.5	88.5
85.1	10.3	43.7	31.0	85.1
79.3	8.0	41.4	29.9	79.3
93.1	10.3	46.0	36.8	93.1
85.1	10.3	46.0	28.7	85.1
86.2	9.2	42.5	34.5	86.2
93.1	10.3	44.8	37.9	93.1
80.5	8.0	41.4	31.0	80.5
96.6	9.2	46.0	41.4	96.6

89.7	10.3	41.4	37.9	89.7
81.6	10.3	43.7	27.6	81.6
82.8	8.0	44.8	29.9	82.8
87.4	9.2	41.4	36.8	87.4
93.1	9.2	46.0	37.9	93.1
88.5	8.0	43.7	36.8	88.5
89.7	10.3	44.8	34.5	89.7
95.4	9.2	44.8	41.4	95.4
94.3	10.3	43.7	40.2	94.3
100.0	11.5	46.0	42.5	100.0
98.9	11.5	44.8	42.5	98.9
88.5	9.2	46.0	33.3	88.5
87.4	10.3	44.8	32.2	87.4
85.1	9.2	46.0	29.9	85.1
88.5	10.3	41.4	36.8	88.5
97.7	9.2	46.0	42.5	97.7
97.7	11.5	46.0	40.2	97.7
93.1	9.2	46.0	37.9	93.1
90.8	9.2	44.8	36.8	90.8
81.6	9.2	42.5	29.9	81.6
89.7	10.3	43.7	35.6	89.7
